# Antioxidant Effect of Soymilk Fermented by *Lactobacillus plantarum* HFY01 on D-Galactose-Induced Premature Aging Mouse Model

**DOI:** 10.3389/fnut.2021.667643

**Published:** 2021-05-17

**Authors:** Chong Li, Yang Fan, Shuang Li, Xianrong Zhou, Kun-Young Park, Xin Zhao, Huazhi Liu

**Affiliations:** ^1^Chongqing Collaborative Innovation Center for Functional Food, Chongqing University of Education, Chongqing, China; ^2^Chongqing Engineering Research Center of Functional Food, Chongqing University of Education, Chongqing, China; ^3^Chongqing Engineering Laboratory for Research and Development of Functional Food, Chongqing University of Education, Chongqing, China; ^4^Department of Clinical Nutrition, Daping Hospital, Army Medical University (Third Military Medical University), Chongqing, China; ^5^College of Biological and Chemical Engineering, Chongqing University of Education, Chongqing, China; ^6^First Affiliated Hospital of Gannan Medical University, Ganzhou, China

**Keywords:** *Lactobacillus plantarum*, antioxidant, D-galactose, fermented soymilk, isoflavone

## Abstract

The antioxidant effect of soymilk fermented by *Lactobacillus plantarum* HFY01 (screened from yak yogurt) was investigated on mice with premature aging induced by D-galactose. *In vitro* antioxidant results showed that *L. plantarum* HFY01-fermented soymilk (LP-HFY01-DR) had better ability to scavenge the free radicals 1,1-diphenyl-2-picrylhydrazyl (DPPH) and 2,2′-azino-bis (3-ethylbenzthiazoline-6-sulphonic acid) diammonium salt (ABTS) than unfermented soymilk and *Lactobacillus bulgaricus*-fermented soymilk. Histopathological observation showed that LP-HFY01-DR could protect the skin, spleen and liver, reduce oxidative damage and inflammation. Biochemical results showed that LP-HFY01-DR could effectively upregulate glutathione (*GSH*), catalase (*CAT*), superoxide dismutase (*SOD*), and glutathione peroxidase (*GSH-Px*) levels and decrease malondialdehyde (*MDA*) content in the liver, brain, and serum. Real-time quantitative reverse transcription polymerase chain reaction further showed that LP-HFY01-DR could promote the relative expression levels of the genes encoding for cuprozinc superoxide dismutase (*Cu/Zn-SOD, SOD1*), manganese superoxide dismutase (*Mn-SOD, SOD2*), *CAT, GSH*, and *GSH-Px* in the liver, spleen, and skin. High-performance liquid chromatography results revealed daidzin, glycitin, genistin, daidzein, glycitein, and genistein in LP-HFY01-DR. In conclusion, LP-HFY01-DR could improve the antioxidant capacity in mice with premature aging induced by D-galactose.

## Impact Statement

*Lactobacillus plantarum* HFY01 is a newly discovered lactic acid bacterium with probiotic potential. The fermentation of soymilk with *L. plantarum* HFY01 can convert more soybean isoflavones into active soybean isoflavones, which are easily absorbed, thereby improving the physiological activity of fermented soymilk. This study found that the soymilk fermented by *Lactobacillus plantarum* HFY01 has a good inhibitory effect on D-galactose-induced premature aging mice via antioxidation.

## Introduction

Oxidation is a necessary process for most living organisms, but most oxidation causes damage to the body. When oxidation occurs in the human body, free radicals are produced in the body. Free radicals are extremely unstable due to their lone electrons, which act on other stable molecules, turning those molecules into free radicals, as well (1, 2). If the cycle continues, many free radicals will be produced, and the body will eventually be damaged (3). Free radicals mainly damage lipids, carbohydrates, proteins, and nucleic acids (4). Some human diseases are related to free radicals, and although the antioxidant system exists in everyone's body, oxidative damage caused by free radicals cannot be completely resisted or repaired (5). There has thus been great effort to research and develop natural antioxidants (6).

D-galactose, which is an aging agent, has been widely used in the construction of cell and animal models of premature aging, but its mechanism is not fully understood. Excessive intake of D-galactose can lead to cellular metabolism disorders, enzyme activity changes, and increased peroxides, leading to oxidative damage to the structure and function of biological macromolecules, which then leads to systemic inflammatory responses and various complications (7, 8).

*Lactobacillus* are common bacteria in daily life, and they are especially important for fermented foods. Human life activities are closely related to the normal physiological functions of *Lactobacillus* (9). These bacteria have many health functions, such as improving nutrition, reducing cholesterol content in serum, enhancing antioxidant and anti-radiation effects, reducing intestinal endotoxin levels, inhibiting the reproduction of spoilage bacteria in the gastrointestinal tract, enhancing the body's immune capacity, preventing the invasion and colonization of pathogenic bacteria in the gastrointestinal tract, and preventing and inhibiting the occurrence of tumors (10, 11). There are many sources of *Lactobacillus* in nature, and since ancient times, *Lactobacillus* applications have been extensive. The antioxidant capacity of these bacteria has recently been a hot topic in the development of *Lactobacillus* products (12). These bacteria have good antioxidant effects, but different *Lactobacillus* species have different antioxidant capacities, which may be due to different concentrations and types of antioxidant components (13). *Lactobacillus* plantarum is different from other *Lactobacillus* species, as *L. plantarum* can produce a large amount of acid, leading to unstable pH in water, and many of these bacteria are live (14). *L. plantarum* has many functions, such as regulating chronic intestinal diseases, regulating immune function, and regulating spirits (15).

A special product of Tibetan areas in China is naturally fermented yak yogurt, which is fermented by a variety of *Lactobacillus*. Yak yogurt has a variety of health care functions and nutritional value. *Lactobacillus* in yak yogurt can regulate normal intestinal flora, inhibit the growth and development of intestinal pathogenic bacteria, inhibit the production of metabolites, prevent intestinal diseases, maintain homeostasis of the body, lower cholesterol levels, and produce anti-aging, anti-tumor, and anti-cancer effects (16–18).

Soybeans have an antioxidant function involving a variety of antioxidant components, among which isoflavones and phenolic substances have the main effect; they help delay aging, reduce cholesterol levels, regulate blood lipid levels, etc. (19–21). In soymilk fermentation, microorganisms decompose protein in the milk and change soybean isoflavone structures. Unique products result from the recombination and decomposition of macromolecules during protein hydrolysis. Moreover, antinutritional substances are decomposed by *Lactobacillus* so that the nutrients in soymilk can be fully utilized by the human body, and fermented soymilk has a stronger antioxidant effect than unfermented soymilk (22).

Here, the *L. plantarum* HFY01 (LP-HFY01) used in this study is a novel *L. plantarum* with good antioxidant activity isolated from naturally fermented yak yogurt from Tibetan areas. LP-HFY01 was used to ferment the soymilk, which was given to mice with premature aging induced by D-galactose. Serum, liver, spleen, brain, and skin oxidative stress levels were assessed to evaluate the antioxidant capacity of LP-HFY01-fermented soymilk and provide theoretical support for the subsequent development of *Lactobacillus*-fermented soybean products.

## Materials and Methods

### Experimental Strains and Reagents

LP-HFY01 was isolated from traditionally fermented yak yogurt (Hongyuan County, Aba Tibetan and Qiang Autonomous Prefecture, Sichuan Province, China). After isolation, purification, and identification, the strain was stored in the China General Microbiological Culture Collection Center (CGMCC, Beijing, China), preservation number 16629. *Lactobacillus bulgaricus* (LB) was purchased from the China Center for Type Culture Collection (CCTCC, Wuhan, China), preservation number AB200048. Vitamin C and D-galactose were purchased from the China National Pharmaceutical Group Corporation (Beijing, China).

### Activation and Treatment of LP-HFY01 and LB

LP-HFY01 and LB were inoculated into MRS (DeMan-Rogosa-Sharpe) liquid medium at a 2% inoculation level and then cultured and activated for two generations (using a 37°C constant temperature water bath shaker). The MRS liquid medium containing the bacteria after activation for was centrifuged at 4,000 r/min for 10 min. The upper medium was removed, and 0.9% normal saline of the same volume was added to prepare the bacterial suspension.

### Preparation of Fermented Soymilk

The selected soybeans (Heilongjiang Province, China) were soaked in double-distilled water at a ratio of 1:2 for 12 h. Then, the soaked soybeans and double-distilled water at a ratio of 1:8 were ground in a soymilk machine and filtered with sterile gauze. The soymilk was divided into sterile conical bottles and sterilized in an autoclave at 121°C for 15 min. The two kinds of *Lactobacillus* were inoculated into the sterilized soymilk at a 2% inoculation level. The soymilk was then fermented at 37°C for 12 h to get two kinds of fermented soymilk.

### *In vitro* Evaluation of the Antioxidant Capacity of Fermented Soymilk

A 0.1 mol/L 1,1-diphenyl-2-picrylhydrazyl (DPPH) solution was prepared with absolute ethanol and stored in the dark. In addition, the Vitamin C (V_C_) aqueous solution was prepared as a control. Next, 2 mL of the test sample aqueous solution + 2 mL of the DPPH solution were added to the sample test tube, shaken well, and placed in the dark at room temperature for 30 min. This mixture was then added to a 96-well plate, and the absorbance at 517 nm (A_1_) was measured; additionally, the absorbance of 2 mL of the DPPH solution + 2 mL of ultrapure water (A_0_) and the absorbance of 2 mL of the test sample solution + 2 mL of absolute ethanol (A_2_) were measured (23). The following formula was used: SC (%) = [1-(A_1_-A_2_)/A_0_] × 100%.

A 2,2′-azino-bis(3-ethylbenzthiazoline-6-sulphonic acid) diammonium salt (ABTS) free radical working solution was prepared as follows: add 3 mg of ABTS to 0.8 mL of ultrapure water, mix, and dissolve to create solution A; add 1 mg of potassium persulfate to 1.5 mL of ultrapure water, mix, and dissolve to create solution B; take 0.2 mL of solution A and 0.2 mL of solution B, mix, and place in the dark for 12 h to form stable free radical ions. In a 5-mL test tube, 1 mL of the ABTS free radical working solution and 0.4 mL of the test sample aqueous solution were added, and the test tube was placed in the dark for 10 min. The absorbance at 734 nm was measured; parallel measurements were performed three times, and the average value was calculated (24). The following formula was used: SC (%) = [1-(A_i_-A_j_)/A_f_] × 100%. A_i_ denotes the absorbance value of the ABTS working solution + the test sample solution; A_j_ denotes the blank correction, or the absorbance value of the test sample solution + absolute ethanol; and A_f_ denotes the blank control, or the absorbance value of the ABTS working solution + ultrapure water. The value of SC_50_ (the concentration of the sample required to scavenge 50% DPPH or ABTS free radicals) is used to reflect the antioxidant activity of the sample.

### Grouping of Mice and Induction of Premature Aging

Sixty-six-week-old female Kunming mice (purchased from the animal experiment center of Chongqing Medical University) were fed adaptively in the animal room for 1 week. During this period, they were fed a normal diet and given drinking water. After 1 week, the mice were randomly divided into six groups, with 10 mice in each group: (1) the LP-HFY01-fermented soymilk group (LP-HFY01-DR), (2) the premature aging model group, (3) the normal control group, (4) the LB-fermented soymilk group (LB-DR), (5) the unfermented soymilk group (NF-DR), and (6) the V_C_ group. The specific operations were as follows: the premature aging model group and normal control group mice were gavaged with 0.2 mL of 0.9% normal saline daily throughout the experimental period; the V_C_ group mice were gavaged with 0.2 mL of V_C_ (100 mg/kg) daily throughout the experimental period; and the LB-DR, NF-DR, and LP-HFY01-DR group mice were gavaged with 0.2 mL of LB-fermented soymilk, unfermented soymilk, and LP-HFY01-fermented soymilk, respectively, daily throughout the experimental period. Starting on the third week of the experimental period, all mice except for the normal control group mice were injected with 0.2 mL of D-galactose (120 mg/kg) solution daily. The experimental period was 8 weeks.

### Collection of Experimental Samples

At the end of the experiment, blood was collected from the eyeballs of the mice and centrifuged (3,000 r/min) with a high-speed freezing centrifuge for 10 min. The supernatant was carefully poured into a clean centrifuge tube to obtain serum, which was then placed in an ultra-low temperature refrigerator (−80°C) for cryopreservation. The mice were euthanized by decapitation, and their brain, liver, spleen, and skin tissues were dissected. Brain tissue was directly frozen in a sealed bag. Liver, spleen, and skin tissues were placed in normal saline (which was refrigerated at 4°C in advance) to wash away the blood, and some tissues were soaked in formalin (10%, v/v), while the remaining tissues were frozen (−80°C) in sealed bags.

### Histological Analysis of the Liver, Spleen, and Skin

Some tissues and organs were fixed with 10% formalin for 24 h, cut longitudinally, embedded in paraffin, and stained with hematoxylin-eosin (H&E), Masson's trichrome, and toluidine blue. The sections were studied under a microscope (BX43, Olympus, Tokyo, Japan). Image-Pro Plus 6.0 (Media Cybernetics, Maryland, USA) was used to quantitatively analyze the significance of different pictures.

### Determination of Antioxidant-Related Indexes in the Liver, Brain, and Serum

Brain tissue (0.1 g) and liver tissue (0.1 g) were weighed, then normal saline (which was refrigerated at 4°C in advance) was added to each at a respective ratio of 1:9. The tissues were put into separate homogenate tubes for grinding. The homogenate was gathered and centrifugated at 2,500 r/min for 10 min. The supernatant from the centrifuged sample was poured into a clean centrifuge tube. The total protein content in the tissue was determined by using the Coomassie brilliant blue method. Catalase (*CAT*) activity, glutathione (*GSH*) content, superoxide dismutase (*SOD*) activity, glutathione peroxidase (*GSH-Px*) activity, and malondialdehyde (*MDA*) content in the serum, liver tissue, and brain tissue of the mice were determined according to the instructions of an experimental kit purchased from the Nanjing Jiancheng Bioengineering Institute (Nanjing, China).

### qPCR Analysis of mRNA in Mice Tissue

Liver, spleen, or skin tissue (100 mg) and 1 mL of Trizol homogenate were transferred into a new 1-mL centrifuge tube with 200 μL of chloroform. This was mixed and left to stand at 4°C for 5 min, then frozen and centrifuged at 14,000 r/min and 4°C for 20 min. The supernatant was drawn into a new 1-mL centrifuge tube; isopropanol in the same volume as the supernatant was added. This was mixed and left to stand at 4°C for 15 min, then frozen and centrifuged at 14,000 r/min and 4°C for 20 min to obtain yellow RNA precipitate. Then, a 75% ethanol solution (75 μL ethanol + 25 μL diethylpyrocarbonate (DEPC) water) was added to the yellow RNA precipitate to wash it. It was then frozen and centrifuged at 14,000 r/min and 4°C for 15 min. The supernatant was poured out, and the centrifuge tube was opened to dry for 3 to 5 min. Then, 20 μL of DEPC water filtered with a 0.22-μL filter was added. This was mixed to obtain total RNA. Then, 1 μL of the RNA stock solution was pipetted into 49 μL of DEPC water. This was mixed to determine the concentration and purity of the RNA. Using the enzyme-free water control, 10 μL of RNA diluent was added in sequence. When the ratio of A260 to A280 was between 1.8 and 2.0, the RNA purity was considered appropriate, and the next step of the experiment could be performed. The RNA stock solution was diluted to 1 μg/mL, and then 1 μL of the RNA template, 1 μL of Oligo (dT) Primer, and 10 μL of RNase-free water were mixed and reacted in a gradient polymerase chain reaction (PCR) instrument at 65°C for 5 min. Then, 1 μL of RiboLock RNase inhibitor, 4 μL of 5× Reaction Buffer, 1 μL of RevertAid M-MuLV, and 2 μL of 10 mM deoxynucleotide (dNTP) mix were added. After mixing, this was put into a gradient PCR instrument at 42°C for 60 min and at 70°C for 5 min to reverse transcribe the RNA into cDNA. Using the cDNA as a template for PCR-specific amplification, 1 μL of the cDNA, 10 μL of master mix, 1 μL of upstream and downstream primers, and 7 μL of double-distilled water were added. This was then mixed and placed into a quantitative PCR (qPCR) instrument. Reaction conditions were: pre-denaturation: 95°C, 30 sec; PCR reaction: 95°C, 5 s and 60°C, 30 s; 40 cycles (25). The gene for β-actin was used as the internal reference to detect the threshold cycle (C_T_) value of the sample. Each sample was subjected to three parallel experiments, and the average value of the three parallel experiments was calculated. The mRNA expression of the target gene was calculated according to the formula 2^−ΔΔCt^. The relevant primer sequences are shown in Table 1.

**Table 1:** Primer sequences.

**Primer**	**Forward primer (3′ to 5′)**	**Reverse primer (3′ to 5′)**
SOD2	CAGACCTGCCTTACGACTATGG	CTCGGTGGCGTTGAGATTGTT
SOD1	AACCAGTTGTGTTGTCAGGAC	CCATGTTTTCTTAGAGTGAGG
GSH-Px	GTCGGTGTATGCCTTCTCGG	AGAGAGACGCGACATTCTCAAT
GSH	TATCAGAGGCGGGAAATCTCTT	ATTCTTGCTTCGGCCACATAC
CAT	GGAGGCGGGAACCCAATAG	GTGTGCCATCTCGTCAGTGAA
β-actin	GGCATCACACTTTCTACAACG	GGCAGGAACATTAAAGGTTTC

*SOD1, Cu/Zn-SOD, cuprozinc-superoxide dismutase; SOD2, Mn-SOD, manganese superoxide dismutase; CAT, catalase; GSH, glutathione; GSH-Px, glutathione peroxidase*.

### HPLC Analysis of Active Components in Fermented and Unfermented Soymilk

Daidzin, daidzein, glycitin, glycitein, genistein, and genistin were all obtained from Beijing Putian Tongchuang Biotechnology Co., Ltd. (Beijing, China). These six standard products were dissolved with 70% ethanol. After that, a mixed standard solution of 50 μg/mL was prepared. The fermented and unfermented soymilk were lyophilized into powder, dissolved in 70% ethanol, and mixed into 2 mg/mL of extraction solution. After centrifugation (4°C; 10,000 r/min; 10 min), the supernatant was filtered through an organic 0.22-μm filter. High-performance liquid chromatography (HPLC) conditions were: UltiMate3000 HPLC system (Thermo Fisher Scientific); 5-μL injection volume; and Accucore C18 column (2.6 μm, 4.6 × 150 mm). The flow rate was 0.5 mL/min, the column temperature was 30°C, and the detection wavelength was 254 nm. The elution conditions are shown in Table 2.

**Table 2:** Gradient elution conditions for high-performance liquid chromatography.

**Time (Min)**	**0.5% acetic acid water (%)**	**Acetonitrile (%)**
−10	85	15
0	85	15
28	65	35
39	58	42
40	85	15

### Statistical Analysis

The data were analyzed by using SPSS 17.0 software. One-way analysis of variance was used to analyze whether there was a statistical difference between the data of each group (*P* < 0.05).

## Results

### *In vitro* Antioxidant Capacity Analysis

**Figure 1:**
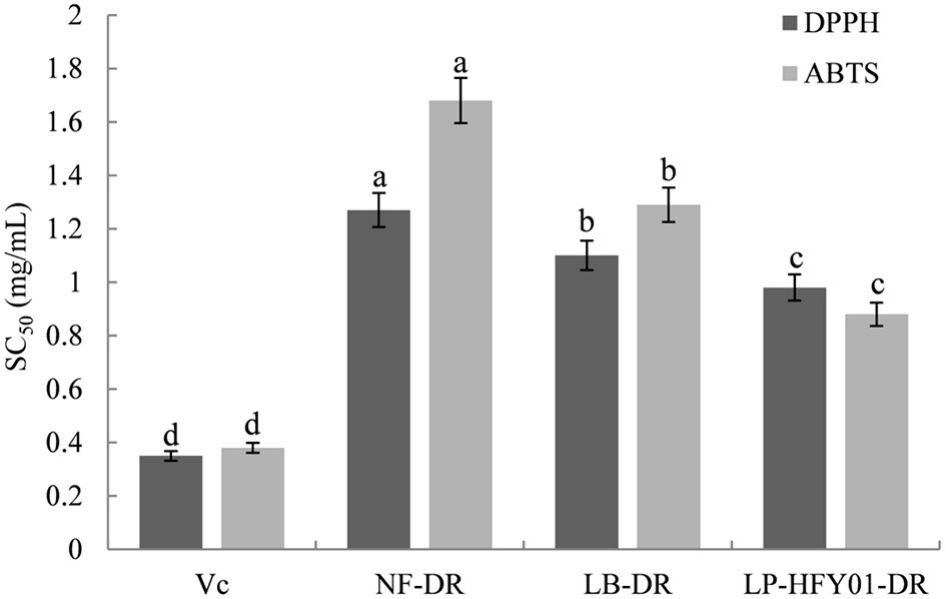
Free radical scavenging ability of NF-DR, LB-DR, and LP-HFY01-DR for DPPH and ABTS. Vc was used as a positive reference. SC_50_: the concentration of the sample required to scavenge 50% DPPH or ABTS free radicals. (a–d) There was a significant difference between different letters in the same column (*P* < 0.05), per Duncan's multiple range test. DPPH, 1,1-diphenyl-2-picrylhydrazyl; ABTS, 2,2′-azino-bis(3-ethylbenzthiazoline-6-sulphonic acid) diammonium salt; NF-DR, unfermented soymilk; LB-DR, Lactobacillus bulgaricus-fermented soymilk; LP-HFY01-DR, Lactobacillus plantarum HFY01-fermented soymilk.

NF-DR, LB-DR, and LP-HFY01-DR showed significant DPPH and ABTS free radical scavenging activity *in vitro* (Figure 1). The SC_50_ values of LP-HFY01-DR for DPPH and ABTS free radical were 0.98 and 0.87 mg/mL, respectively. In addition, NF-DR and LB-DR resisted DPPH and ABTS free radicals to some extent, but the scavenging effect was weaker compared to LP-HFY01-DR. The SC_50_ values of NF-DR for DPPH and ABTS free radical were 1.27 and 1.68, mg/mL respectively, while the SC_50_ values of LB-DR to DPPH and ABTS were 1.10 and 1.29 mg/mL, respectively.

### Histological Analysis of Liver, Spleen, and Skin Tissues

**Figure 2:**
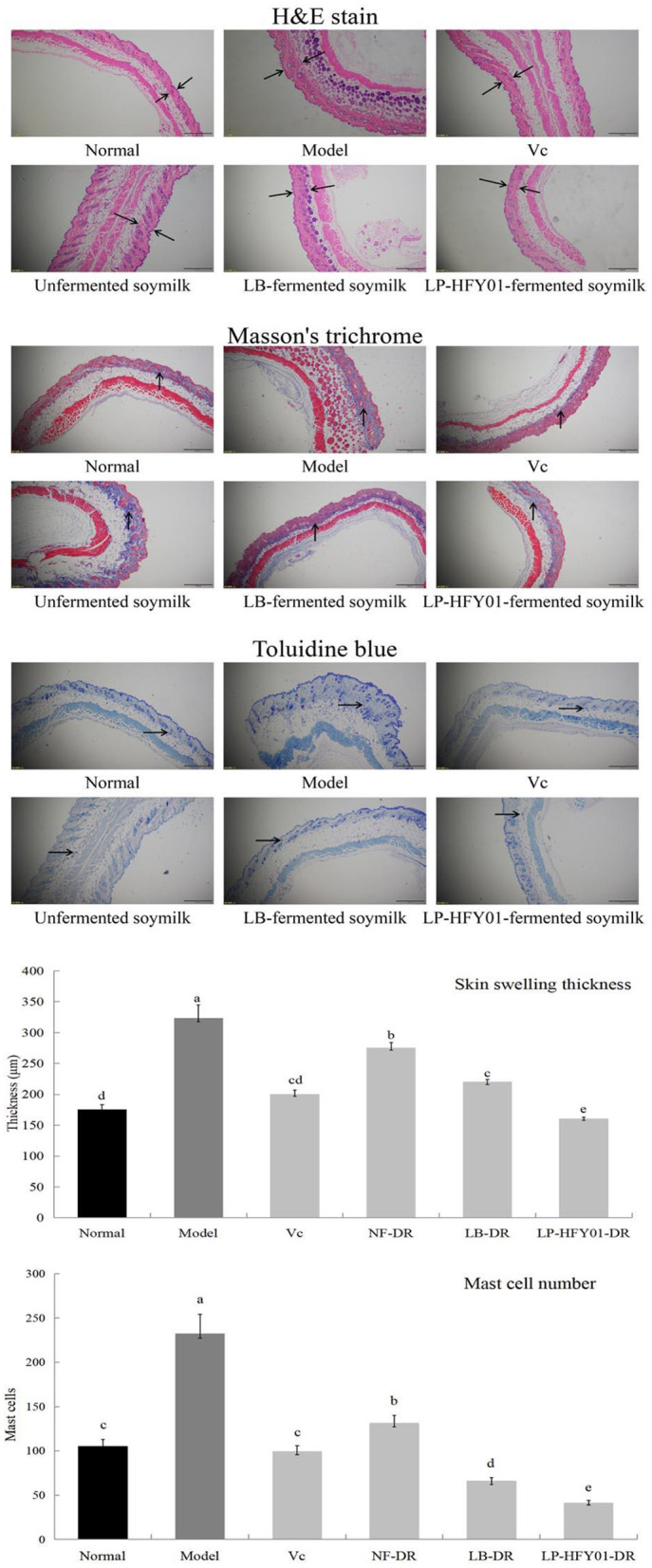
Skin section analysis using H&E, Masson's trichrome, and toluidine blue stains. Normal: No treatment; Model: Premature aging model; V_C_: Treatment with V_C_; Unfermented soymilk: Treatment with unfermented soymilk; LB-fermented soymilk: Treatment with *Lactobacillus bulgaricus*-fermented soymilk; LP-HFY01-fermented soymilk: Treatment with *Lactobacillus plantarum* HFY01-fermented soymilk; H&E: Hematoxylin-eosin; → ←: swelling thickness; ↑: collagen; →: mast cell. (a–e) Mean values with different letters in the same bar graph are significantly different (*P* < 0.05), per Duncan's multiple range test.

As shown by the H&E staining in Figure 2, the epidermis (purple area) and dermis (red area) of the D-galactose-induced premature aging model mice were swollen and thickened. After LP-HFY01-DR treatment, the injury symptoms improved. With Masson's trichrome staining, blue represents the collagen fiber content in the dermis. When the collagen cross-linked, the skin appeared wrinkled and showed other signs of premature aging. The epidermis of the premature aging model group was thicker than that of the normal group, and the epidermis became thinner after LP-HFY01-DR treatment. Toluidine blue stains mast cells (blue and purple dots). Mast cells are the source of inflammatory reactions, thereby showing a positive correlation with inflammatory levels. The thickness of skin swelling and the number of mast cells in the premature aging model group were greater than in the other groups, but the swelling thickness and inflammation cell number were reduced by varying degrees in the VC, NF-DR, LB-DR, and LP-HFY01-DR groups, these results all suggested that LP-HFY01-DR treatment had the best effect on D-galactose-induced skin injury in mice.

**Figure 3:**
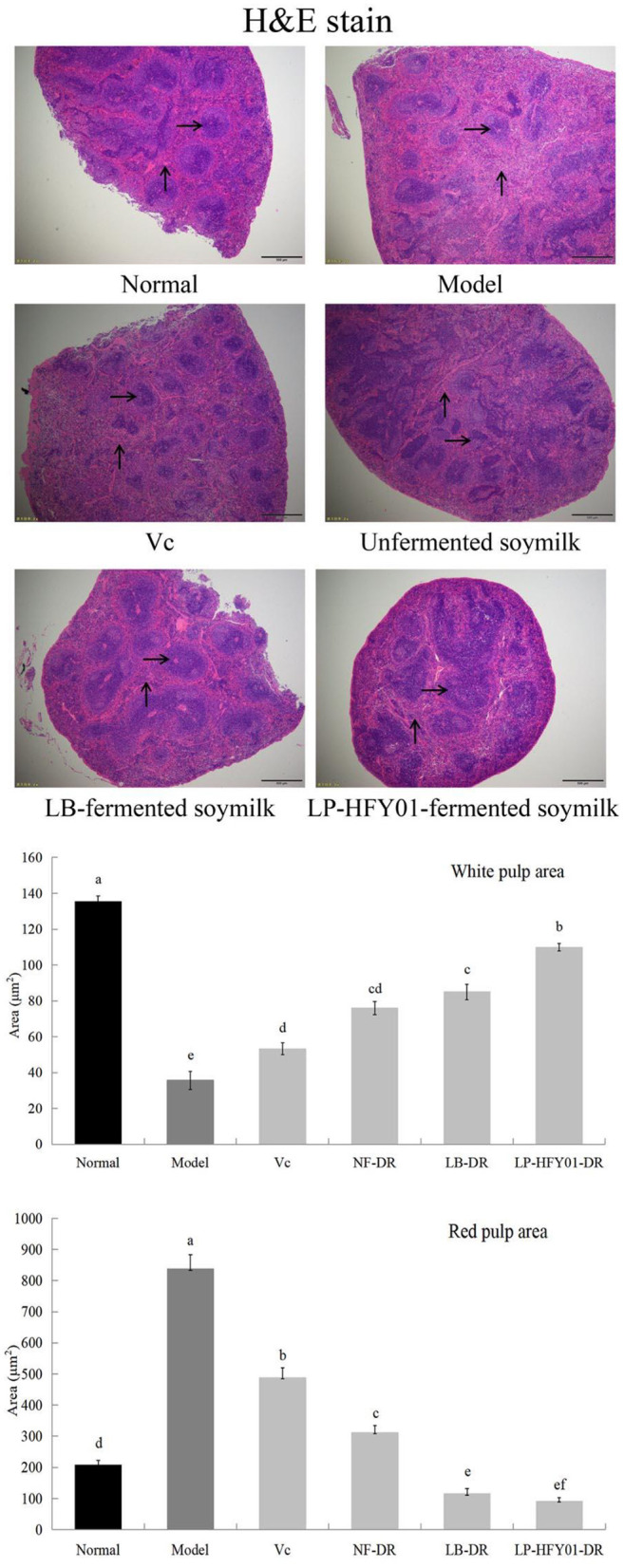
Spleen section analysis using hematoxylin-eosin stain. Normal: No treatment; Model: Premature aging model; V_C_: Treatment with V_C_; Unfermented soymilk: Treatment with unfermented soymilk; LB-fermented soymilk: Treatment with Lactobacillus bulgaricus-fermented soymilk; LP-HFY01-fermented soymilk: Treatment with Lactobacillus plantarum HFY01-fermented soymilk. →: White pulp; ↑: Red pulp. (a–f) Mean values with different letters in the same bar graph are significantly different (*P* < 0.05), per Duncan's multiple range test.

To further evaluate the therapeutic effects of LP-HFY01-DR on mice with premature aging, histopathological liver and spleen changes were observed by using H&E staining (Figure 3). Splenocytes in the normal group were intact, with clear structure and orderly arrangement. On the contrary, the spleen of the mice with premature aging showed obvious damage, with disordered structure, mixed red and white pulp, increased red blood cells, and decreased white pulp lymphocytes. In the V_C_ and NF-DR groups, these characteristics were partially improved, but they were significantly improved in the LP-HFY01-DR and LB-DR groups; specifically, damage was significantly reduced, spleen congestion was reduced, and the boundary between white and red pulp was clear. Results from quantitative analysis of white pulp and red pulp of the spleen showed that the red pulp area in the premature aging model group significantly increased and the white pulp area decreased, but there was significant improvement after LB-DR and LP-HFY01-DR treatment.

**Figure 4:**
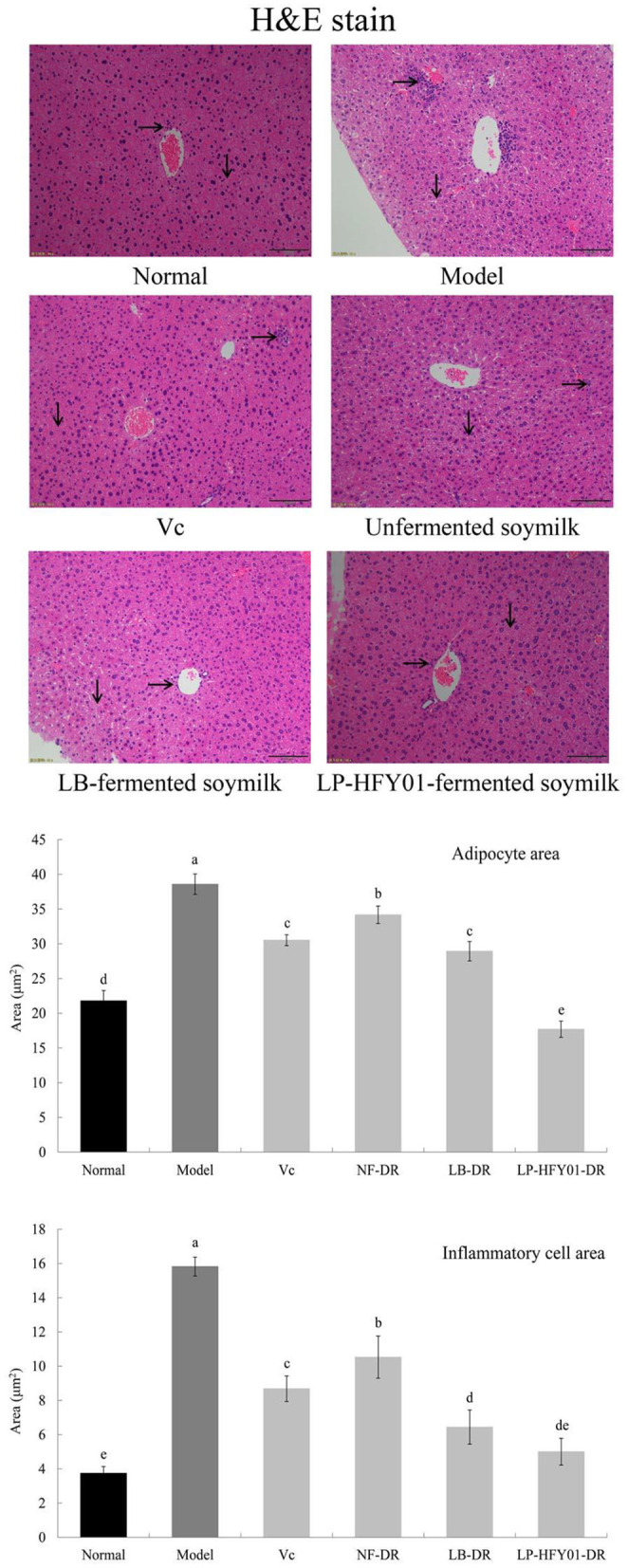
Liver section analysis using hematoxylin-eosin stain. Normal: No treatment; Model: Premature aging model; V_C_: Treatment with V_C_; Unfermented soymilk: Treatment with unfermented soymilk; LB-fermented soymilk: Treatment with *Lactobacillus bulgaricus*-fermented soymilk; LP-HFY01-fermented soymilk: Treatment with *Lactobacillus plantarum* HFY01-fermented soymilk. →: inflammatory cell; ↓: adipocyte. (a–e) Mean values with different letters in the same bar graph are significantly different (*P* < 0.05), per Duncan's multiple range test.

Normal hepatocytes were distributed radially around the central vein, with normal structure (Figure 4). However, in the premature aging model group, the hepatocyte arrangement was disordered, the structure was destroyed, inflammatory cell infiltration was observed, cell necrosis was increased, and hepatocyte steatosis was observed. V_C_, NF-DR, LB-DR, and LP-HFY01-DR alleviated hepatic sinusoid and hepatic plate disorder, alleviated cell necrosis, and reduced inflammatory cell infiltration. LB-DR and LP-HFY01-DR had the best effects, with findings closer to those of the normal group. Histological observation of liver tissue and quantitative analysis of adipocytes and the inflammatory cell area showed that LP-HFY01-DR could improve D-galactose-induced oxidative damage of organs.

### Antioxidant Biochemical Index Analysis

The *MDA* content levels in the liver tissues of the NF-DR, LP-DR, and LP-HFY01-DR groups were 14.91 ± 0.55, 12.88 ± 0.73, and 10.50 ± 0.57 nmol·mL^−1^, respectively (Table 3). This was significantly lower than in the premature aging model group (*P* < 0.05). The *MDA* content levels in the brain tissues and serum of each treatment group were lower than in aging model group, the *MDA* content in the LP-HFY01-DR groups of the brain and serum decreased significantly (*P* < 0.05), with values of 36.29 ± 1.99 and 3.74 ± 0.15 nmol·mL^−1^, respectively. The *MDA* content levels in the serum, liver tissue, and brain tissue of the premature aging model group were 6.52 ± 0.34, 24.45 ± 1.03, and 50.91 ± 1.3 nmol·mL^−1^, respectively. After different treatments, the *MDA* content levels in the brain, liver, and serum of the V_C_, NF-DR, LP-HFY01-DR, and LB-DR groups were decreased, suggesting that V_C_, unfermented soymilk, LP-HFY01-fermented soymilk, and LB-fermented soymilk were beneficial in reducing *MDA* content in mice. The results showed that soymilk fermented by LP-HFY01 was more beneficial than soymilk fermented by LB for reducing *MDA* content in the liver tissue, brain tissue, and serum.

**Table 3:** Effects of soymilk fermented by different strains on MDA content in the liver tissue, brain tissue, and serum of mice.

**Group**	**Liver**	**Brain**	**Serum**
	**MDA/nmol·mL^**−1**^**	**MDA/nmol·mL^**−1**^**	**MDA/nmol·mL^**−1**^**
Normal	13.68 ± 0.66^cd^^A^	31.69 ± 1.54^f^	4.73 ± 0.13^c^
Model	24.45 ± 1.03^a^	50.91 ± 1.3^a^	6.52 ± 0.34^a^
V_C_	19.57 ± 1.06^b^	42.32 ± 0.96^c^	4.67 ± 0.21^c^
NF-DR	14.91 ± 0.55^c^	45.81 ± 1.51^b^	5.55 ± 0.38^b^
LB-DR	12.88 ± 0.73^d^	39.21 ± 1.19^d^	4.38 ± 0.30^c^
LP-HFY01-DR	10.50 ± 0.57^e^	36.29 ± 1.99^e^	3.74 ± 0.15^d^

A *Mean value ± standard deviation*.

a−f *Mean values with different letters in the same column are significantly different (P < 0.05), per Duncan's multiple range test. NF-DR, unfermented soymilk; LB-DR, Lactobacillus bulgaricus-fermented soymilk; LP-HFY01-DR, Lactobacillus plantarum HFY01-fermented soymilk; MDA, malondialdehyde*.

As seen in Table 4, the *GSH* content in the serum, liver and brain tissues of the LP-HFY01-DR and LB-DR groups was significantly higher than in the premature aging model group (*P* < 0.05), and the *GSH* content in the liver of the LP-HFY01-DR group was significantly higher than that of the other groups (*P* < 0.05). The *GSH* content levels in the serum of the NF-DR groups were close to those of the premature aging model group, and the improvement effect was weak. The results showed that V_C_, unfermented soymilk, LB-fermented soymilk, and LB-HFY01-fermented soymilk were helpful to increase the *GSH* content in mice, while LB-HFY01-fermented soymilk had the best effect.

**Table 4:** Effects of soymilk fermented by different strains on GSH content in the liver tissue, brain tissue, and serum of mice.

**Group**	**Liver**	**Brain**	**Serum**
	**mgGSH/gprot**	**mgGSH/gprot**	**mgGSH/L**
Normal	33.78 ± 1.44^a^^A^	21.36 ± 1.03^a^	10.56 ± 0.19^a^
Model	8.61 ± 1.15^ef^	11.91 ± 1.47^e^	7.51 ± 0.23^d^
Vc	10.25 ± 1.26^d^	16.48 ± 0.44^cd^	8.43 ± 0.18^bc^
NF-DR	10.65 ± 0.86^d^	15.43 ± 0.39^d^	7.64 ± 0.41^d^
LB-DR	21.71 ± 1.49^c^	17.32 ± 0.28^bc^	8.30 ± 0.25^c^
LP-HFY01-DR	28.47 ± 0.87^b^	18.59 ± 0.41^b^	8.77 ± 0.13^b^

A *Mean value ± standard deviation*.

a−f *Mean values with different letters in the same column are significantly different (P < 0.05), per Duncan's multiple range test. NF-DR, unfermented soymilk; LB-DR, Lactobacillus bulgaricus-fermented soymilk; LP-HFY01-DR, Lactobacillus plantarum HFY01-fermented soymilk; GSH, glutathione*.

*SOD* activity levels in the liver tissue, brain tissue, and serum of the premature aging model group were 177.67 ± 9.42 U·mg^−1^ prot, 162.88 ± 8.04 U·mg^−1^ prot, and 126.31 ± 11.84 U·mL^−1^, respectively (Table 5). After treatment with unfermented soymilk, V_C_, LB-fermented soymilk, and LP-HFY01-fermented soymilk, *SOD* activity showed different degrees of improvement, but the *SOD* activity levels in the liver tissue, serum, and brain tissue after LP-HFY01-fermented soymilk treatment were closest to those of the normal group (*P* < 0.05).

**Table 5:** Effects of soymilk fermented by different strains on SOD activity in the liver tissue, brain tissue, and serum of mice.

**Group**	**Liver**	**Brain**	**Serum**
	**SOD/U·mg^**−1**^ prot**	**SOD/U·mg^**−1**^ prot**	**SOD/U·mL^**−1**^**
Normal	268.25 ± 14.20^b^^A^	361.66 ± 20.58^a^	193.88 ± 10.05^a^
Model	177.67 ± 9.42^e^	162.88 ± 8.04^e^	126.31 ± 11.84^c^
Vc	213.2 ± 9.20^d^	267.69 ± 27.62^c^	178.51 ± 11.99^ab^
NF-DR	225.13 ± 7.04^d^	216.76 ± 36.10^d^	164.62 ± 7.42^b^
LB-DR	289.45 ± 5.79^a^	254.84 ± 14.16^cd^	161.74 ± 4.98^b^
LP-HFY01-DR	245.09 ± 12.70^c^	316.70 ± 8.29^b^	195.82 ± 12.49^a^

A *Mean value ± standard deviation*.

a−e *Mean values with different letters in the same column are significantly different (P < 0.05), per Duncan's multiple range test. NF-DR, unfermented soymilk; LB-DR, Lactobacillus bulgaricus-fermented soymilk; LP-HFY01-DR, Lactobacillus plantarum HFY01-fermented soymilk; SOD, superoxide dismutase*.

Table 6 shows that compared with the premature aging model group, the *CAT* activity levels in the liver tissues of the LB-DR and LP-HFY01-DR groups were significantly increased (130.87 ± 4.11 and 143.93 ± 13.59 U·mg^−1^ prot, respectively), and the difference was significant (*P* < 0.05). *CAT* activity in the brain tissues of LP-HFY01-DR group was significantly greater than in the premature aging model group (*P* < 0.05), while *CAT* activity changes in the V_C_ and LB-DR groups were similar. The serum *CAT* activity levels in the LP-HFY01-DR and LB-DR groups were 86.70 ± 3.13 and 72.46 ± 1.33 U·mL^−1^, respectively. Compared with the premature aging model group, *CAT* activity was significantly increased in the LP-HFY01-DR and LB-DR groups (*P* < 0.05). Soymilk fermented by LP-HFY01 was therefore shown to improve *CAT* activity in the liver tissue, serum, and brain tissue of mice.

**Table 6:** Effects of soymilk fermented by different strains on CAT content in the liver, serum and brain tissue of mice.

**Group**	**Liver**	**Brain**	**Serum**
	**CAT/U·mg^**−1**^ prot**	**CAT/U·mg^**−1**^ prot**	**CAT/U·mL^**−1**^**
Normal	164.21 ± 12.20^a^^A^	86.05 ± 3.59^a^	103.37 ± 2.26^a^
Model	99.98 ± 2.09^d^	61.71 ± 1.37^e^	60.27 ± 2.09^f^
Vc	121.64 ± 4.38^c^	75.31 ± 1.67^bc^	78.29 ± 1.63^c^
NF-DR	120.19 ± 5.06^c^	68.55 ± 0.45^d^	65.66 ± 2.29^e^
LB-DR	130.87 ± 4.11^bc^	72.36 ± 0.84^c^	72.46 ± 1.33^d^
LP-HFY01-DR	143.93 ± 13.59^b^	78.24 ± 1.25^b^	86.70 ± 3.13^b^

A *Mean value ± standard deviation*.

a−f *Mean values with different letters in the same column are significantly different (P < 0.05), per Duncan's multiple range test. NF-DR, unfermented soymilk; LB-DR, Lactobacillus bulgaricus-fermented soymilk; LP-HFY01-DR, Lactobacillus plantarum HFY01-fermented soymilk; CAT, catalase*.

Table 7 shows that compared with the premature aging model group, GSH-Px activity levels in the liver, brain, and serum of the NF-DR and V_C_ groups were increased, and the differences were significant (*P* < 0.05). GSH-Px activity levels in the serum, liver tissue, and brain tissue in the LP-HFY01-DR group were 736.86 ± 20.05 U·mL^−1^, 1700.23 ± 83.63 U·mg^−1^ prot, and 712.67 ± 5.81 U·mg^−1^ prot, respectively, and they were higher than in the other groups (except for in the brain tissue and serum of the normal group). GSH-Px activity levels in the liver and brain tissues of the LB-DR group were similar to those of the LP-HFY01-DR group, but GSH-Px activity in the serum of the LB-DR group was significantly different from that of the LP-HFY01-DR group. The results showed that the effect of LB-fermented soymilk on GSH-Px activity was slightly lower than that of LP-HFY01-fermented soymilk, and GSH-Px activity was highest with LP-HFY01-fermented soymilk.

**Table 7:** Effects of soymilk fermented by different strains on GSH-Px content in the liver tissue, brain tissue, and serum of mice.

**Group**	**Liver**	**Brain**	**Serum**
	**GSH-Px/U·mg^**−1**^**	**GSH-Px/U·mg^**−1**^**	**GSH-Px/U·mL^**−1**^**
	**prot**	**prot**	
Normal	850.63 ± 34.33^c^^A^	724.17 ± 9.83^a^	946.66 ± 17.65^a^
Model	723.15 ± 26.88^d^	642.83 ± 6.83^d^	569.60 ± 11.88^e^
Vc	1010.72 ± 68.60^b^	706.06 ± 7.82^b^	689.80 ± 9.14^c^
NF-DR	917.4 ± 22.37^bc^	671.49 ± 12.87^c^	627.97 ± 15.54^d^
LB-DR	1659.79 ± 91.12^a^	699.25 ± 6.08^b^	668.23 ± 18.48^c^
LP-HFY01-DR	1700.23 ± 83.63^a^	712.67 ± 5.81^ab^	736.86 ± 20.05^b^

A *Mean value ± standard deviation*.

a−e *Mean values with different letters in the same column are significantly different (P < 0.05), per Duncan's multiple range test. NF-DR, unfermented soymilk; LB-DR, Lactobacillus bulgaricus-fermented soymilk; LP-HFY01-DR, Lactobacillus plantarum HFY01-fermented soymilk; GSH-Px, glutathione peroxidase*.

### Antioxidant-Related Gene Expression in the Liver, Skin, and Spleen

**Figure 5:**
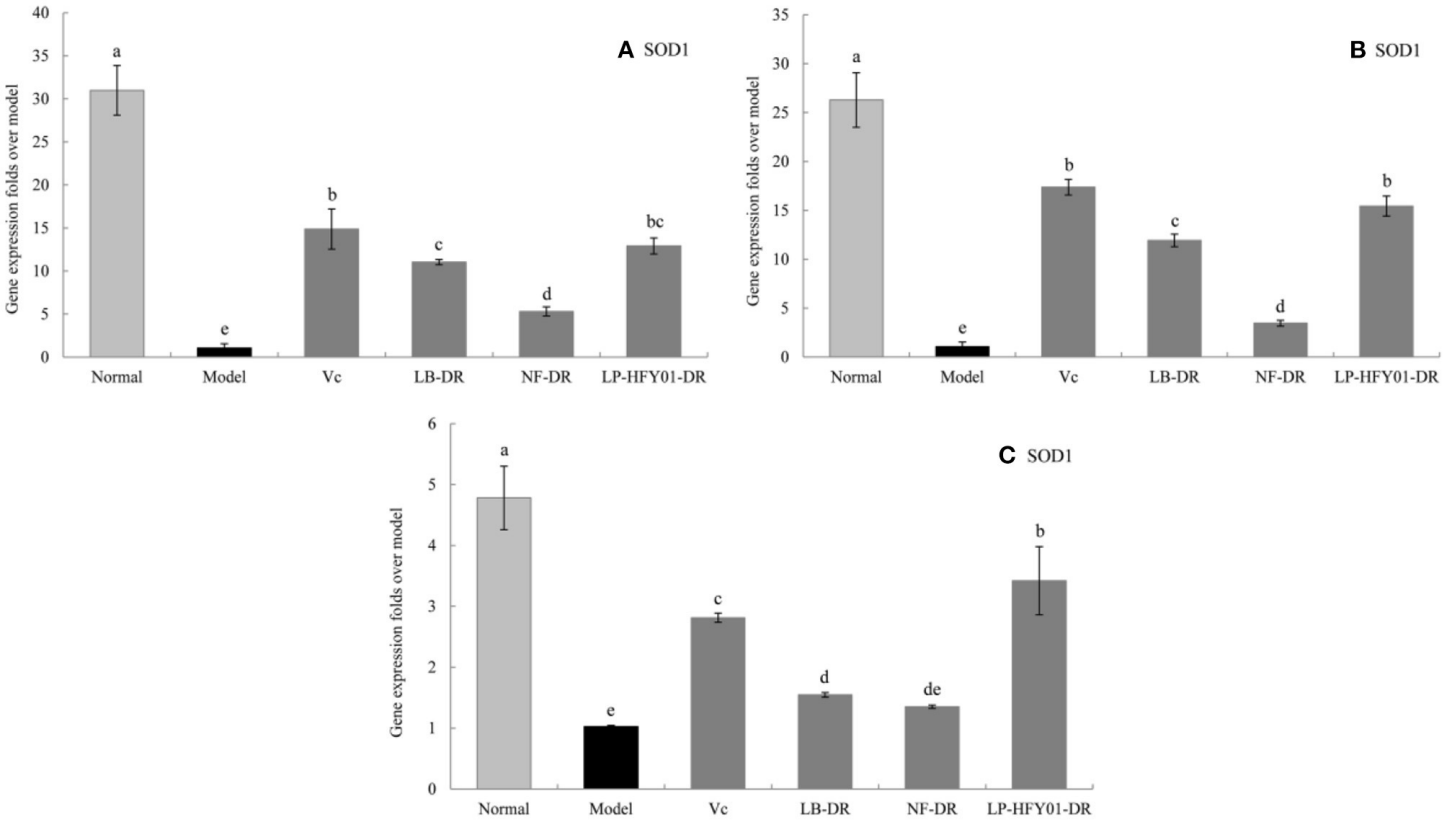
Gene expression of SOD1 in the liver, skin, and spleen. (a–e) Mean values with different letters in the same bar graph are significantly different (*P* < 0.05), per Duncan's multiple range test. **(A)** Liver SOD1 expression; **(B)** Skin SOD1 expression; **(C)** Spleen SOD1 expression.

**Figure 6:**
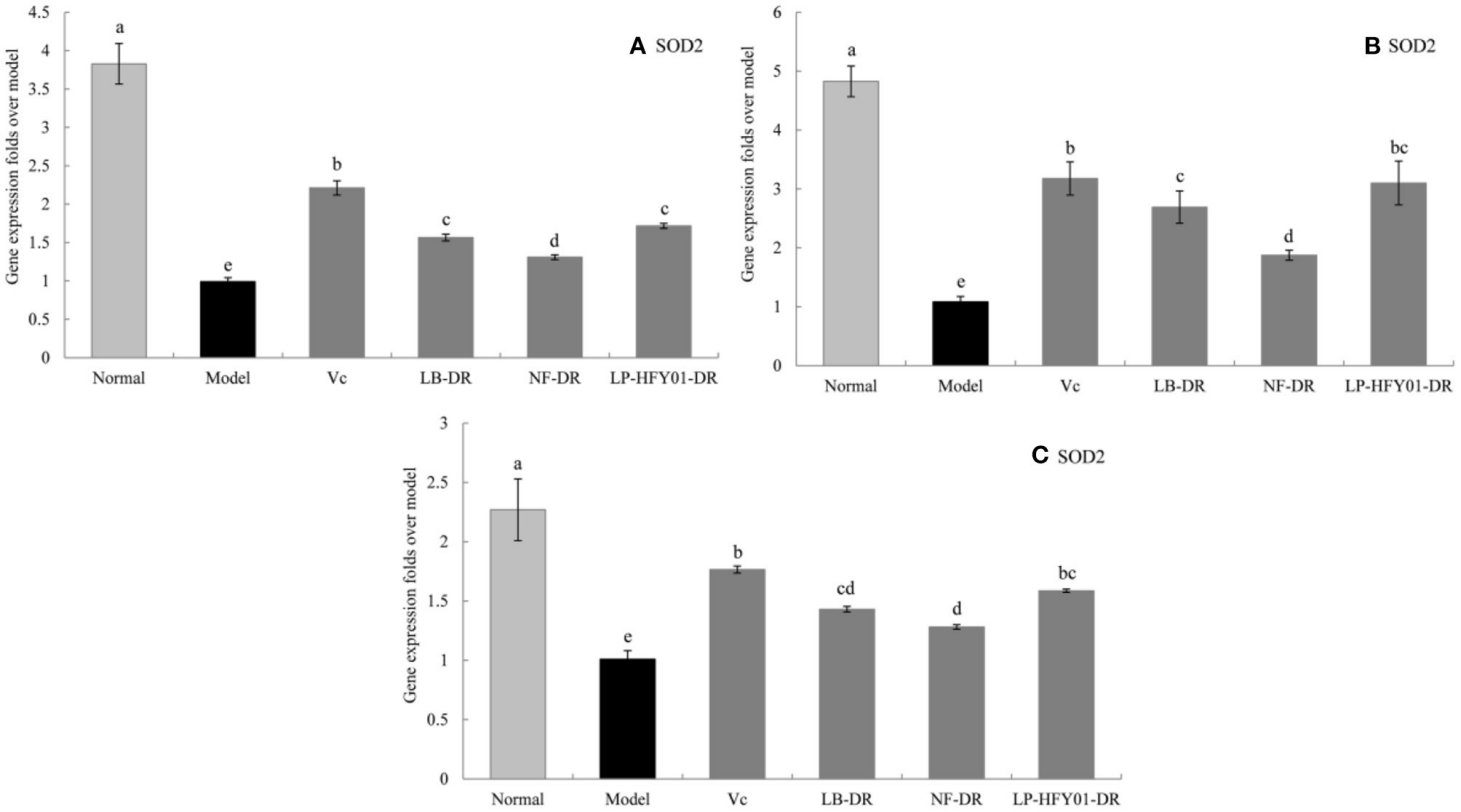
Gene expression of SOD2 in the liver, skin, and spleen. (a–e) Mean values with different letters in the same bar graph are significantly different (*P* < 0.05), per Duncan's multiple range test. **(A)** Liver SOD2 expression; **(B)** Skin SOD2 expression; **(C)** Spleen SOD2 expression.

**Figure 7:**
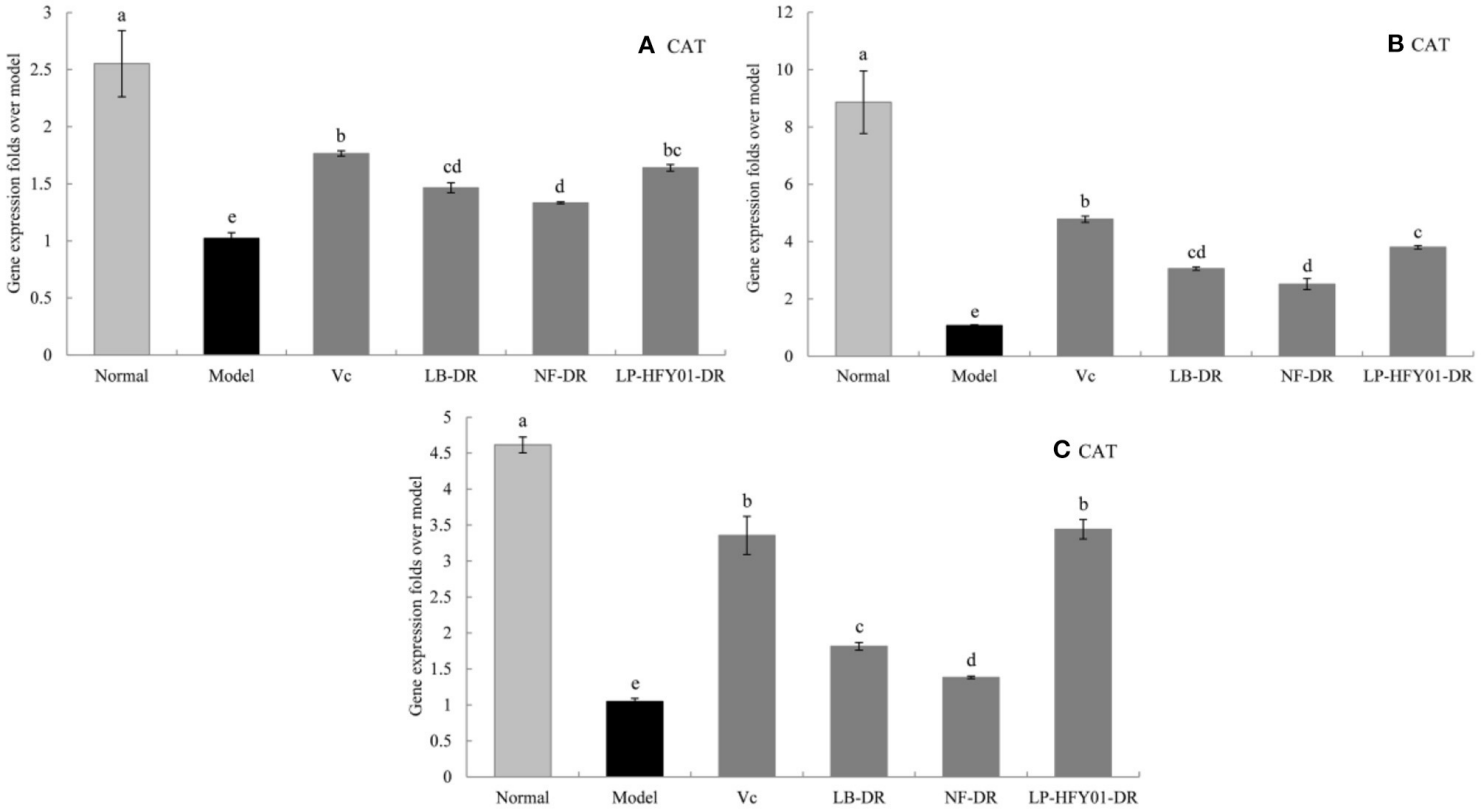
Gene expression of CAT in the liver, skin, and spleen. (a–e) Mean values with different letters in the same bar graph are significantly different (*P* < 0.05), per Duncan's multiple range test. **(A)** Liver CAT expression; **(B)** Skin CAT expression; **(C)** Spleen CAT expression.

**Figure 8:**
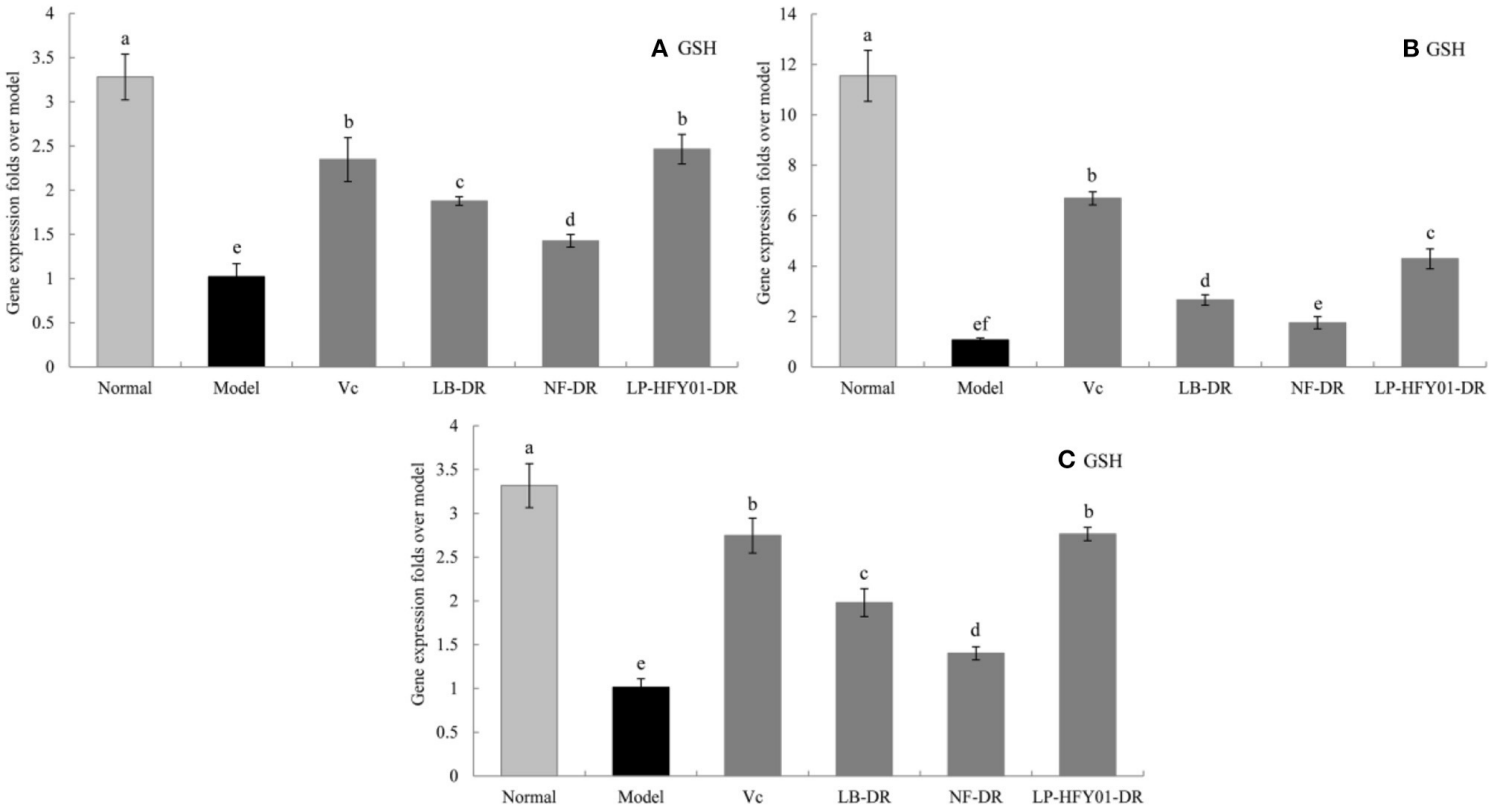
Gene expression of GSH in the liver, skin, and spleen. (a–f) Mean values with different letters in the same bar graph are significantly different (*P* < 0.05), per Duncan's multiple range test. **(A)** Liver GSH expression; **(B)** Skin GSH expression; **(C)** Spleen GSH expression.

**Figure 9:**
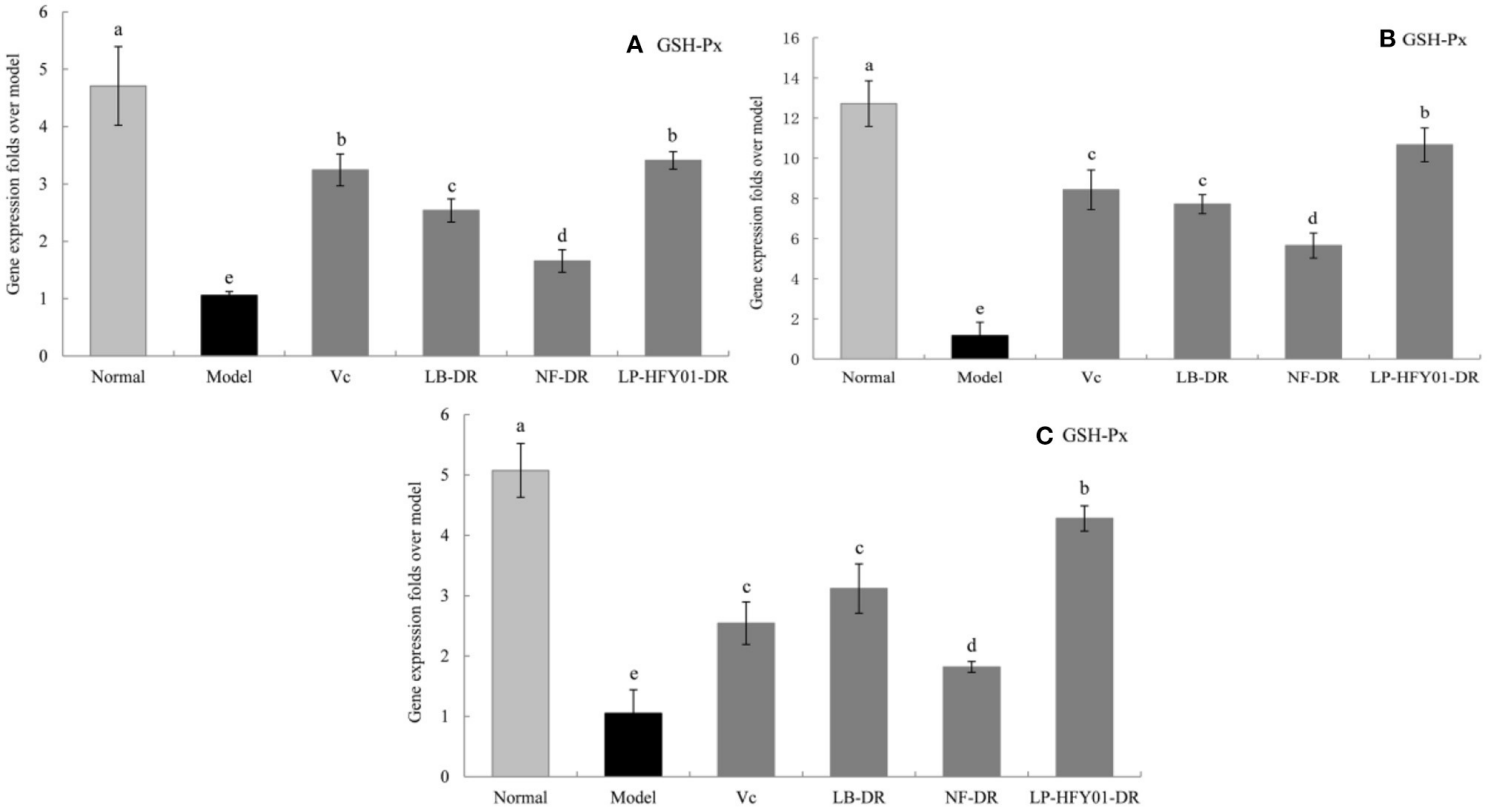
Gene expression of GSH-Px in the liver, skin, and spleen. (a–e) Mean values with different letters in the same bar graph are significantly different (*P* < 0.05), per Duncan's multiple range test. **(A)** Liver GSH-Px expression; **(B)** Skin GSH-Px expression; **(C)** Spleen GSH-Px expression.

To further explore the antioxidant mechanism of LP-HFY01-DR, the relative gene expression levels of *SOD1, SOD2, CAT, GSH*, and *GSH-Px* in the liver, spleen, and skin (Figures 5–9) were detected by using real-time quantitative reverse transcription PCR (qRT-PCR). The levels of *SOD1, SOD2, CAT, GSH*, and *GSH-Px* in the liver, spleen, and skin of the normal group were strongest. The levels of *SOD1, SOD2, CAT, GSH*, and *GSH-Px* in the treatment groups (V_C_, NF-DR, LB-DR, and LP-HFY01-DR) were stronger than in the premature aging model group but weaker than in the normal group. In the treatment groups, the expression levels of antioxidant-related genes (*SOD1, SOD2, CAT, GSH*, and *GSH-Px*) in the liver, spleen, and skin of the LB-DR and LP-HFY01-DR groups were higher than in the NF-DR group. In addition, the expression levels of antioxidant-related genes in the LP-HFY01-DR group were higher than in the LB-DR group, especially for the GSH-Px-encoding gene (*P* < 0.05). The results showed that LP-HFY01-DR could effectively improve the expression of antioxidant-related genes in the liver, spleen, and skin of mice with premature aging induced by D-galactose.

### Analysis of Isoflavones in Fermented and Unfermented Soymilk

**Figure 10:**
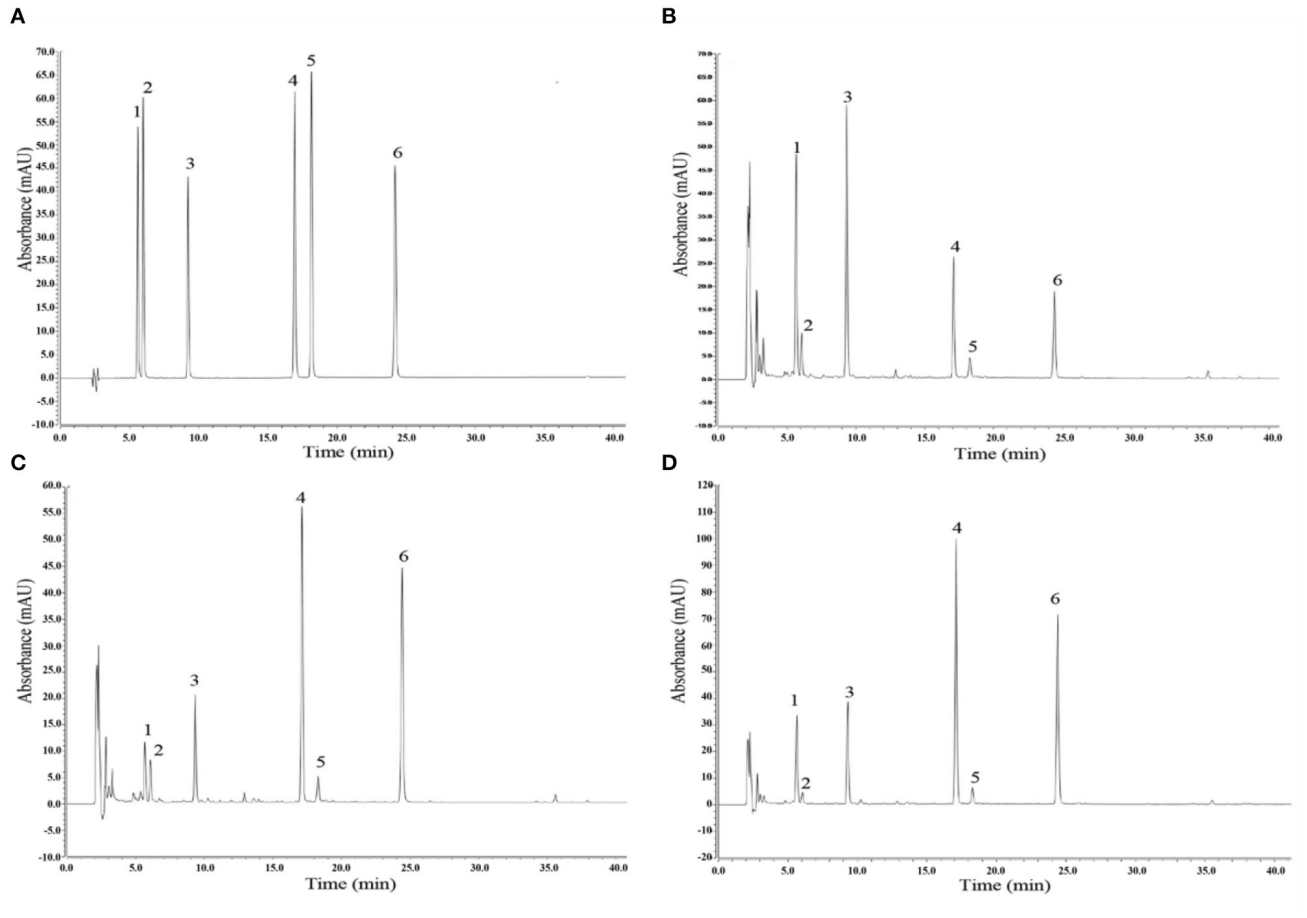
Analysis of isoflavones via high-performance liquid chromatography. **(A)** Mixed standard chromatogram of isoflavones; **(B)** Chromatogram of unfermented soymilk; **(C)** Chromatogram of *Lactobacillus bulgaricus*-fermented soymilk; **(D)** Chromatogram of *Lactobacillus plantarum* HFY01-fermented soymilk. Peak 1: daidzin; peak 2: glycitin; peak 3: genistin; peak 4: daidzein; peak 5: glycitein; peak 6: genistein.

Compared with the standard HPLC chromatogram (Figure 10A), six isoflavones were identified in unfermented soymilk (Figure 10B), LB-fermented soymilk (Figure 10C), and LP-HFY01-fermented soymilk (Figure 10D): daidzin (peak 1), glycitin (peak 2), genistin (peak 3), daidzein (peak 4), glycitein (peak 5), and genistein (peak 6). The daidzin and genistin content decreased, while the daidzein and genistein content increased after fermentation, which may be due to the role of probiotics in the degradation of the isoflavones.

## Discussion

Oxidation caused by free radicals is the main inducer of human aging, so attention has been given to the evaluation of antioxidant levels and antioxidant-related products. DPPH is an artificially synthesized, stable organic free radical, and its methanol or ethanol solution presents a dark purple-red color. After pairing its lone electrons, DPPH is reduced to a yellow non-free radical form, and its fading degree has a quantitative relationship with the number of electrons accepted (26). ABTS, which is a chromogenic agent, can be oxidized to green ABTS^+^ under the action of a suitable oxidant. When antioxidants are added, ABTS·^+^ production is inhibited, so antioxidant activity can be judged by measuring absorbance at specific wavelengths (27). We used V_C_ as the positive control and detected the scavenging ability of LP-HFY01-DR, LB-DR, and NF-DR on DPPH and ABTS to preliminarily determine the antioxidant capacity of each substance to provide a reference for following experiments. *In vitro* antioxidant experiment results showed that LP-HFY01-DR had the strongest scavenging ability on DPPH and ABTS.

D-galactose, which is a natural reducing sugar, is a normal metabolite in animals. Under normal conditions, lactose is hydrolyzed into glucose and galactose *in vivo*, and galactose is hydrolyzed into glucose in the liver. Excessive D-galactose can cause increased metabolic rates in the body and more oxidative accumulation of free radicals, thus leading to damage to DNA, proteins, cell membrane lipids, and other macromolecules in cells, resulting in mitochondrial damage, nerve damage, and increased cognitive decline, thereby making the body age (28). D-galactose-induced premature aging animal models are similar to natural aging in terms of physiological and biochemical indicators, so they are widely used in drug screens and evaluations of anti-aging food products (29).

The liver plays an important role in regulating homeostasis of the body (growth and development, disease resistance, and energy supply). The metabolic activities of the liver include anabolism, energy metabolism, catabolism, and carbohydrate metabolism. After digestion, food is decomposed into glucose and transported to the liver, and it is absorbed by the body after metabolism by the liver. Drugs are also metabolized by the liver, and blind intake of drugs may cause damage to the liver (30). The spleen plays an important role in the digestive, immune, and blood systems. White blood cells in the spleen are an important source of antibodies, which can resist infection, phagocytize pathogenic bacteria, and promote wound healing. The spleen can recover and store the iron used to make new red blood cells, which can be quickly replenished and released by the human body in a state of blood loss. The spleen also plays an important role in filtering harmful bacteria in the blood and managing and regulating blood volume (31). Oxidative damage of the skin is a key sign of aging. The main skin components are protein, fat, carbohydrates, water, and electrolytes. Protein is the main component of collagen, which constitutes the epidermis and dermis. Oxidative stress induced by D-galactose can destroy protein, increase collagen cross-linking, reduce collagen sensitivity, reduce collagen decomposition, and reduce elasticity and toughness. Previous studies using sections of skin stained with H&E, Masson's trichrome, and toluidine blue have shown that LP-HFY01-DR can reduce swelling and thickening of the skin and inhibit oxidative damage of the skin caused by D-galactose by reducing oxidative stress levels and protein cross-linking in skin tissue (32, 33). In addition, brain atrophy in mice with premature aging is more obvious than atrophy of other organs, and brain aging is a characteristic of systemic aging. Long-term injection of D-galactose causes oxidative stress, which leads to mitochondrial damage, neurodegenerative deformation, and cognitive decline in mice. LP-HFY01-DR can inhibit oxidative damage in brain tissue by improving the overall antioxidant capacity of the body (34).

The body can resist cell injury induced by long-term, low-dose injections of D-galactose through the actions of antioxidant enzymes and non-enzyme antioxidant components *in vivo* (35). SOD is a metal enzyme that exists widely in organisms. It has good thermal stability and acid-base stability, and it can catalyze the conversion of superoxide ions into oxygen and hydrogen peroxide. SOD can be divided into three types of metal cofactors: Cu/Zn-SOD, Mn-SOD, and Fe-SOD. As a eukaryotic enzyme, Cu/Zn-SOD (blue-green) exists in the cytoplasm of eukaryotic cells, with Cu^2+^ and Zn^2+^ as active centers; Mn-SOD (purple) mainly exists in prokaryotic cells and the eukaryotic matrix (such as in mitochondria), with Mn^4+^ as an active center; and Fe-SOD (yellowish brown) mainly exists in prokaryotic cells and a few plants (36, 37). When endogenous and exogenous ROS increased, intracellular SOD1 translocated rapidly to the nucleus, maintaining the stability of the nuclear genome. H_2_O_2_ promotes direct translocation of SOD1 to the nucleus, regulates the binding of SOD1 to dun1 through the effectors of Mec1/ATM and Dun1/Cds1 kinases, and phosphorylates S60 and s99 of SOD1 (38). In a variety of cell lines and inflammatory tissues, SOD1 regulates the activities of TNF-α, NF-κB, MAPK, JNK, Akt, AP-1, and JAK-STAT through ROS signaling pathway (39). Manganese superoxide dismutase encoded by SOD2 can scavenge superoxide and produce hydrogen peroxide and oxygen. It is found that peroxide and superoxide can induce the transcription of SOD2 gene, and the inhibition of manganese superoxide dismutase on tumor cells may also lead to the decline of tumor cells through the up regulation of p53 (40). In this study, LP-HFY01-DR upregulated SOD levels in the liver, serum, and brain of mice with premature aging induced by D-galactose. Furthermore, LP-HFY01-DR increased the mRNA expression of Cu/Zn-SOD and Mn-SOD in the liver, spleen, and skin of oxidative mice, and it further repaired oxidative damage of the liver, spleen, and brain and improved the symptoms of skin peroxidation. The regulation of SOD1 and SOD2 by the sample may affect the inflammation and cancer pathways, and some diseases have preventive and control effects. The in-depth mechanism needs to be further studied. CAT is an antioxidant enzyme that mainly exists in the microsomes of red blood cells and other tissues and cells, as well as in mitochondria and plasma. It can decompose hydrogen peroxide into molecular oxygen and water, remove hydrogen peroxide in the body, and prevent cell oxidative damage. In our experiment, we detected increased CAT levels in the liver, spleen, brain, skin, and serum of oxidative mice treated with LP-HFY01-DR, suggesting the synergistic effect of CAT and SOD. SOD can decompose ${\text{O}}_{2}^{-}$ into hydrogen peroxide, and hydrogen peroxide can be further reduced to H_2_O by CAT. At the same time, the oxygen content in cells increases, which has a protective effect in terms of oxidative damage (41). GSH is a low-molecular-weight, non-enzymatic scavenger that has a strong scavenging effect on lipid free radicals. It directly or indirectly participates in a variety of cell activities, and one of its important functions is to cooperate with other related metabolic enzymes to form resistance to oxidative stress. LP-HFY01-DR can reduce the consumption of GSH caused by D-galactose, thus reducing peroxidation damage (42). GSH-Px is a peroxidase that is widely distributed in the body. It is mainly divided into cytoplasmic GSH-Px, plasma GSH-Px, phospholipid hydroperoxide GSH-Px, and gastrointestinal-specific GSH-Px. GSH-Px can catalyze GSH to glutathione disulfide, reduce toxic peroxide to non-toxic hydroxyl compounds, and promote H_2_O_2_ decomposition, thus protecting the structure and function of cell membranes from oxidative damage caused by D-galactose (43, 44). MDA content reflects the level of oxygen free radicals and the intensity of lipid peroxidation. Long-term D-galactose stimulation produces free radicals, leading to lipid peroxidation, and the final product is MDA. MDA causes the polymerization of macromolecules, such as cross-linked proteins and nucleic acids, which is cytotoxic. Therefore, inhibition of MDA activity can protect organs and tissues from oxidative damage (45). In this study, both LB-DR and LP-HFY01-DR increased the levels of SOD, CAT, GSH, and GSH-Px in the serum, liver, and brain and downregulated the levels of MDA, thus reducing damage caused by D-galactose. In addition, LB-DR and LP-HFY01-DR both effectively reduced the mRNA expression of SOD1, SOD2, CAT, GSH, and GSH-Px in the liver, spleen, and skin to reduce oxidative damage, but the effect of LP-HFY01-DR was better than that of LB-DR.

In recent years, with in-depth studies of natural antioxidants, studies and applications of *Lactobacillus* and related products have attracted much attention. As living probiotics, *Lactobacillus* can produce a large amount of lactic acid from fermentable carbohydrates. These bacteria are widely found in humans, livestock, poultry intestines, and fermented foods (46). Most *Lactobacillus* have important physiological functions in the human body. These bacteria can secrete a variety of enzymes, help the body digest food, provide nutrients, and transmit genetic information; further, the adhesion and colonization of these bacteria in the intestinal tract (which forms a physiological barrier) and the lactic acid and acetic acid in synthetic products can reduce pH and redox potential, and the effect can inhibit the growth of harmful bacteria. Therefore, *Lactobacillus* play an important role in nutritional status, physiological functions, immune responses, tumorigenesis, and anti-aging processes (33, 47, 48). The *L. plantarum* bacterium has good antioxidant capacity (49). In this study, LP-HFY01 was selected from fermented yak yogurt in the early laboratory stage, and it had good gastrointestinal tolerance and antioxidant properties *in vitro*. It was found that LP-HFY01-DR could reduce oxidation levels in serum and main tissues and organs, and it significantly improved oxidative damage of the liver, spleen, and brain.

**Figure 11:**
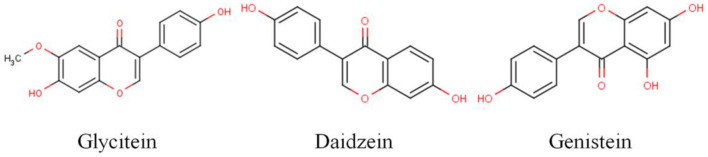
Structure of the three main soybean isoflavones.

Soybean isoflavones are a mixture of polyphenols, which mainly exist in the form of genistein, daidzein, and glycitein (Figure 11). Under natural conditions, most exist in the form of β-glucoside. In recent years, isoflavone glycosides have been found to exhibit acetylation, malonylation, succinylation, and so on, in which genistein, daidzein, and their glycosides play the main physiological functions (50). In this study, soymilk fermented by LP-HFY01 was analyzed by HPLC, and the six identified isoflavones were daidzin, glycitin, genistin, daidzein, glycitein, and genistein. Soybean isoflavones are stable in nature, and it is not easy to destroy isoflavones during cooking, but baking will lead to the loss of some isoflavones (51). Unfermented soybean products mainly exist in the form of glycosides, and fermented soybean products mainly exist in the form of aglycone, which has higher bioavailability, as reflected in this experiment (Figure 10). Soybean isoflavones are good antioxidants and tumor suppressors, especially for hormone-related tumors, such as breast cancer and prostate cancer; they can reduce blood cholesterol levels, coronary atherosclerosis, and peripheral arterial vascular damage, and their weak estrogen-like effect has a certain preventive effect on menopausal syndrome and bone loss in women (52, 53). In addition, soybean isoflavones can improve lipid metabolism, and they can enhance non-specific immunity and anti-inflammatory effects, mainly related to daidzein and genistein (54, 55). Therefore, based on the antioxidant properties of soymilk (soybean isoflavones) and LP-HFY01, the factors described herein have been validated through this study, with soymilk fermented by LP-HFY01 showing high antioxidant capacity, which is helpful to comprehensively inhibit D-galactose-induced premature aging in mice.

LP-HFY01-fermented soymilk improves the flavor of soymilk, increases the nutrients that are beneficial to the body's absorption, and has the dual health functions of soybeans and *Lactobacillus plantarum*, which can be applied to the development of health foods that are beneficial to gastrointestinal absorption (56). However, we have not further studied the anti-premature aging biological activity and specific anti-premature aging mechanism of LP-HFY01-fermented soymilk from the protein level, the change mechanism of biologically active ingredients (such as soybean isoflavones) in the process of LP-HFY01 fermented soymilk, and we will further improve them in future research.

**Figure 12:**
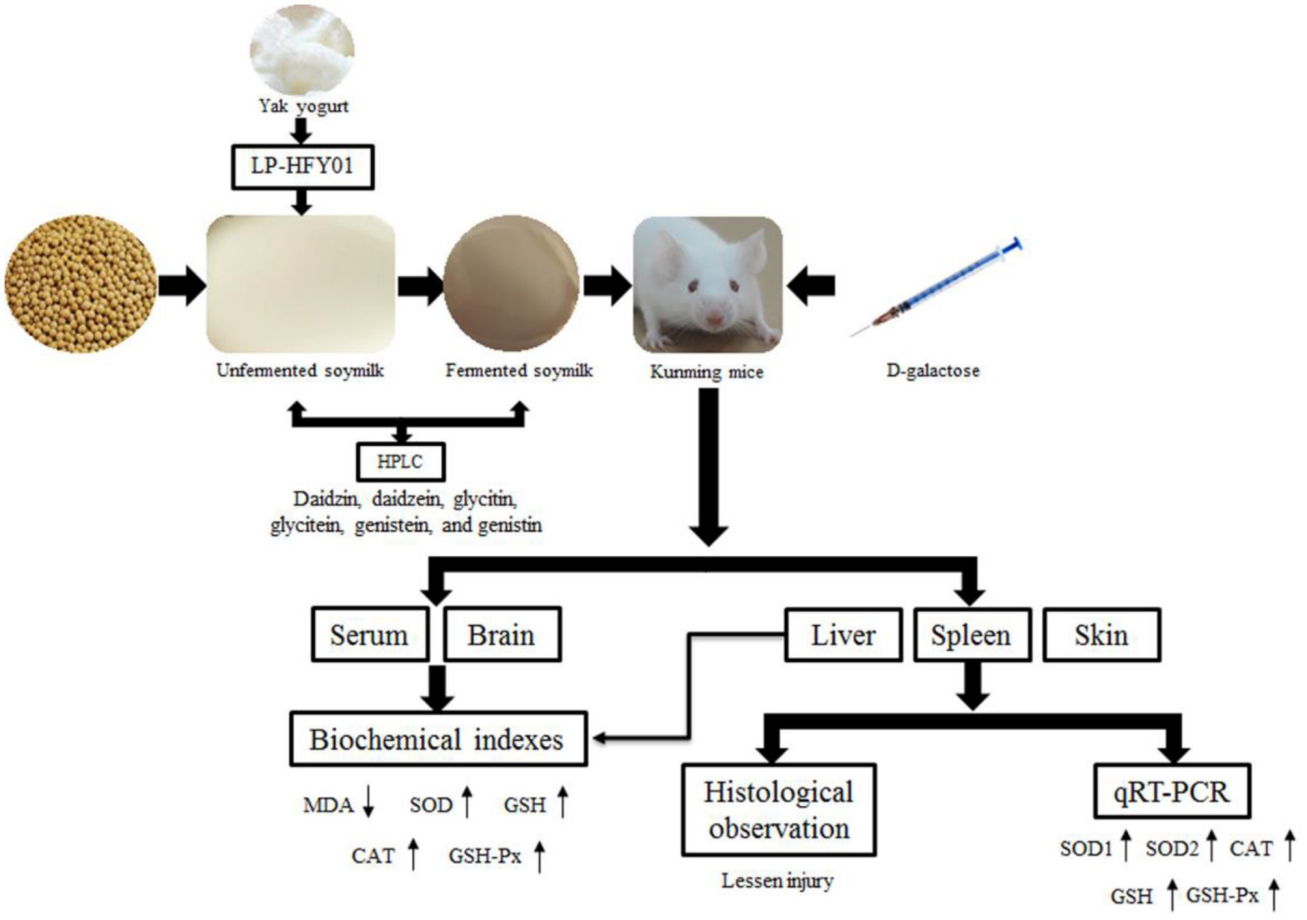
The mechanism of this study. LP-HFY01, Lactobacillus plantarum HFY01; HPLC, high-performance liquid chromatography; MDA, malondialdehyde; SOD, superoxide dismutase; GSH, glutathione; CAT, catalase; GSH-Px, glutathione peroxidase; qRT-PCR, quantitative reverse transcription polymerase chain reaction.

In conclusion, LP-HFY01-fermented soymilk can protect multiple organs from oxidative stress injury; significantly increase GSH, SOD, CAT, and GSH-Px levels in the serum, brain, and liver; and reduce MDA content. Furthermore, LP-HFY01-fermented soymilk can effectively regulate the expression of antioxidant-related genes in the liver, spleen, and skin (promoting the levels of SOD1, SOD2, CAT, GSH, and GSH-Px). In addition, six soybean isoflavones (daidzin, daidzein, glycitin, glycitein, genistein, and genistin) were identified in LP-HFY01-fermented soymilk (Figure 12). Therefore, LP-HFY01-fermented soymilk can significantly benefit D-galactose-induced premature aging in mice, and its antioxidant effect is more obvious than that of LB-fermented soymilk, which has wide application potential.

## Data Availability Statement

The original contributions presented in the study are included in the article/supplementary material, further inquiries can be directed to the corresponding author/s.

## Ethics Statement

The protocol for these experiments was approved by the Ethics Committee of Chongqing Collaborative Innovation Center for Functional Food (201901035B), Chongqing, China.

## Author Contributions

CL and YF performed the majority of the experiments and wrote the manuscript. SL, XZho, and K-YP contributed to the data analysis. XZha and HL designed and supervised the study and checked the final manuscript. All authors contributed to the article and approved the submitted version.

## Conflict of Interest

The authors declare that the research was conducted in the absence of any commercial or financial relationships that could be construed as a potential conflict of interest.
